# Teaching child and adolescent psychiatry to undergraduate medical students - A survey in German-speaking countries

**DOI:** 10.1186/1753-2000-4-21

**Published:** 2010-07-24

**Authors:** Reiner Frank, Florian Frank

**Affiliations:** 1 Clinic for Child and Adolescent Psychiatry, Psychosomatics and Psychotherapy, Ludwig-Maximilians-Universitaet Munich, Lindwurmstrasse 2 a, 80337 Munich, Germany; 2 Innsbruck Medical University, Mitterweg 13, 6040 Innsbruck, Austria

## Abstract

**Objective:**

To conduct a survey about teaching child and adolescent psychiatry to undergraduate medical students in German-speaking countries.

**Methods:**

A questionnaire was sent to the 33 academic departments of child and adolescent psychiatry in Germany, Austria, and the German-speaking part of Switzerland.

**Results:**

All departments responded. For teaching knowledge, the methods most commonly reported were lectures and case presentations. The most important skills to be taught were thought to be how to assess psychopathology in children and how to assess families. For elective courses, the departments reported using a wide range of teaching methods, many with active involvement of the students. An average of 34 hours per semester is currently allocated by the departments for teaching child and adolescent psychiatry to medical students. Required courses are often taught in cooperation with adult psychiatry and pediatrics. Achievement of educational objectives is usually assessed with written exams or multiple-choice tests. Only a minority of the departments test the achievement of skills.

**Conclusions:**

Two ways of improving education in child and adolescent psychiatry are the introduction of elective courses for students interested in the field and participation of child and adolescent psychiatrists in required courses and in longitudinal courses so as to reach all students. Cooperation within and across medical schools can enable departments of child and adolescent psychiatry, despite limited resources, to become more visible and this specialty to become more attractive to medical students. Compared to the findings in earlier surveys, this survey indicates a trend towards increased involvement of academic departments of child and adolescent psychiatry in training medical students.

## Introduction

In a recent review of child and adolescent psychiatry (CAP) in undergraduate medical education, Sawyer et al. [1] identified 18 studies conducted between 1970 and 2007 in the United Kingdom, Europe, the United States, Canada, Japan, Australia, and New Zealand. They found only limited agreement on curricula content. Goals regarded as relevant were evaluating children and families, understanding normal child development, and communication skills. Little time was allocated in the medical school programs for teaching CAP: The average number of teaching hours overall was about 20, with a range of 0 to 439. The authors concluded that undergraduate medical students do not receive enough education in CAP. They recommended promoting national and international standards and encouraging stronger collaboration among teaching staff across different medical schools.

Barriers to teaching CAP include lack of adequate faculty, time, money, and curricular resources [2]. Kálmán et al. [3] in their survey on undergraduate teaching of CAP in European medical schools investigated where and to what extent CAP is taught in Europe, requested information on how teaching is organized, on curriculum content, and on assessment procedures, and discussed future directions and developments aimed at consolidating and enhancing the teaching of the specialty throughout Europe. They found that lectures were the teaching method used most often to mediate knowledge, although teaching in smaller groups was thought to be more effective. "Bedside teaching," "e-learning," and "edited videotapes" were also mentioned by some respondents. The authors' overall impression was of "predominantly theoretical teaching," which provides only limited opportunities for patient contact and the development of any clinical skills with children and families. Participants in the survey said they would welcome opportunities for staff training and exchanges with well-developed teaching centers.

In the survey by Sawyer and Giesen on current practice in Australia [4], participants were asked to rank 8 teaching objectives. The teaching of skills was given the highest priority. Methods used to teach skills were role-plays, working with videotapes, interview training, and contact with real patients.

To gain insight into the practice of CAP, students find it helpful to observe skills demonstrated by teachers. Fine [5] proposed "simulated clinical situations, which can be shown on videotape" as an effective teaching method. Forgotson and Sweeney [6] described their use of edited videotapes to teach child psychiatry to medical students. They employed videos to present an interview as a whole, to show the interviewer's reaction to the interview, to identify elements of nonverbal behavior, and to call attention to behavior that is relevant to differential diagnosis. They proposed that a series of interviews could illustrate a condition better than is possible with a single interview.

Video teaching could be an important component of teaching medical students: It enables exposing students to a greater number of clinical child psychiatry problems than they might otherwise see [7]. Fox [2] pointed out that stimulus videotapes engage the learner on multiple levels, and that information provided in small units has the value of novelty and utility, an emotional value, and an entertainment value.

In **Germany**, the 26 academic departments of CAP have all been in existence for many years. There, as in many other countries, CAP is not a required part of medical training [8]. The German curriculum for medical students (*Approbationsordnung*, Medical Education Act) was revised in 2002 by the national medical licensing board (part of the Federal Ministry of Health). The revision mandated, and has since led to, fundamental changes in medical education. Learning objectives now focus on knowledge and skills useful to primary health care physicians. The new curriculum emphasizes coordination among departments within medical schools regarding the concepts to be taught. So that skills can be learned more effectively, bedside teaching in small groups, problem-based courses, and training in communication skills have been implemented [9-11].

In **Austria**, CAP has been a specialty of its own only since 2007. Independent academic departments exist at 2 of the 4 universities there, one having been established in 1975 (Vienna) and the other in 2008 (Salzburg). In Vienna, there is a stand-alone CAP curriculum.

In **Switzerland**, all 3 medical schools are well established and have a long tradition. In **Switzerland **as in **Germany**, there are detailed catalogues of learning objectives for undergraduate medical training. Priorities in both countries are based on the relevance for diagnosis, therapy, general practice, emergencies, and prevention.

In our own efforts to improve education for medical students, the survey by Sawyer et al. [1] provided the impetus for us to conduct a similar survey in the German-speaking parts of Europe that have medical schools. Our goal in the present study was to assess the current state of education in CAP for undergraduate medical students at German-speaking medical schools.

## Methods

A short questionnaire (see Appendix) was developed based on the review article by Sawyer et al. [1]. The questionnaire was sent to all 33 academic departments of CAP in the German-speaking parts of Europe: 26 in Germany, 4 in Austria, and 3 in Switzerland. After 3 mailings and some personal reminders, the response rate was 100%. Further information was obtained from the descriptions provided by the academic departments on their Web sites.

## Results (Table 1)

**Table 1:** Teaching activities of 33 academic departments of CAP

	n	%
**Educational Objectives**		
		
*Knowledge*		
Diagnosis and treatment of CAP disorders	27	82
Normal development	23	70
Psychopathology and etiology	3	9
Other: Transition to pathology, differential diagnosis, communication skills, doctor-patient interaction, personal and family situation (*1 each*)	1	3
		
*Skills*		
Assessment of psychopathology (child/adolescent)	26	79
Assessment of families	21	64
Communication skills	2	6
History-taking, appropriate medication in accordance with evidence-based medicine, assessment of the family's strengths and resources, identification of biopsychosocial influencing factors (*1 each*)	1	3
		
**Teaching Methods**		
		
*Required courses*	*26*	*79*
Lectures	25	76
Case presentations	21	64
Seminars	11	33
Bedside teaching	4	12
		
*Elective courses*	*7*	*22*
Seminars	7	22
Problem-based learning	4	14
E-learning	3	10
Communication and interaction training	2	6
Video seminars	2	6
Interactive learning	2	6
		
**Collaboration**		
		
Adult psychiatry	24	73
Pediatrics	16	48
Psychosomatic medicine	13	39
Neurology	5	15
Social medicine	2	6
Psychology	2	6
Forensic psychiatry, internal medicine, gynecology, preventive medicine (*1 each*)	1	3
		
**Assessment**		
		
Written examinations	9	27
Multiple-choice tests	8	24
Questions contributed to adult psychiatry examination	6	18
Oral examinations	4	12
Objective structured clinical examination	3	9
No examination	3	9
Not specified	2	6
		
**Hours taught per semester (n = 21)**	Mean	Range
	34	1-212

n = number of departments responding (multiple responses possible)

### Educational Objectives

There was broad agreement among the 33 departments on educational objectives. Knowledge about "diagnosis and treatment of CAP disorders" and about normal child development were considered to be important educational objectives. Skills regarded as important for undergraduate students were the assessment of psychopathology in children and adolescents and the assessment of families.

### Teaching Methods

Of the 33 departments, 26 (79%) were engaged in teaching required courses for medical students. Lectures and case presentations were the teaching methods used most often to convey knowledge and insight into the practice of CAP within the required curriculum. Methods aiming at more active involvement of the students, such as seminars and bedside teaching, were used much less frequently. Where CAP was an elective, skills were taught with more intensive and more participatory teaching methods.

### Hours of CAP Taught per Semester

The mean number of required hours of CAP taught per semester for undergraduate medical students was 34 (range: 1 - 212 hours) based on replies from 21 respondents (64%). Nine respondents (27%) gave imprecise information or none at all. The average time spent in elective courses was 31 hours, based on information from 3 respondents (9%). Of those departments responding, none was involved in both required and elective courses.

### Collaboration with Other Departments

Of the 33 departments, 24 (73%) cooperate mainly with the department of adult psychiatry and 16 (48%) cooperate with pediatrics. Three of the 24 departments have an agreement with adult psychiatry to share teaching activities and to take on the teaching responsibility for up to one third of all students.

### Assessment

Of the 33 departments of CAP, 9 (27%) assess students' knowledge and skills with written tests and another 8 (24%) use multiple-choice tests. Six (18%) contribute questions to the tests given in adult psychiatry, with an average of 4.5 questions being included (range: 2-10). Only a few of the departments assess skills by means of an oral examination or an objective structured clinical examination (OSCE). And a few give no examination at all or did not provide any information about testing. (The total is more than 33 because some departments reported using more than one approach.)

### Using a Combination of Methods to Achieve a Given Learning Objective

In the Heidelberg Curriculum Medicinale a combination of methods is specified for teaching a given topic, here illustrated with the topic of suicidal behavior in children and adolescents [8]. The overall goals are for the students to

• know risk factors and be able to ask relevant questions

• be able to make the necessary decisions

• know and be able to apply legal regulations

• be able to establish contact with a patient in an appropriate and empathic manner and have examined, under supervision, at least one standardized patient.

There are 4 levels at which the students are taught:

Level 1: Knowledge. Here the students have been introduced to the topic, often via a case demonstration.

Level 2: Competencies. The students have learned to arrive at the diagnosis of the disorder, to know what to consider in making the differential diagnosis. They must have a basic understanding of the epidemiology, pathology, clinical picture, diagnosis, and treatment of the disorder.

Level 3: Skills. The students have explored risk factors for suicidal behavior with a standardized patient and have been given feedback.

Level 4: Experience. The students have gained experience and confidence in diagnosing children and adolescents with the disorder.

To achieve the goals outlined earlier, the following methods are combined:

• Lecture "emergencies in CAP" with a connection to the corresponding lecture in adult psychiatry

• Problem-oriented learning: case presentation "suicidal versus self-mutilating behavior"

• Role-play with standardized patient (adolescent or adult)

• E-learning: "suicidality in children and adolescents"

• Video seminar on suicidality in children and adolescents

The amount of teaching time for this package, targeted at all students, is 212 hours within 1 semester.

### Collaboration within and between medical schools and with other institutions

An example of collaboration within a medical school is a seminar in cooperation with pediatricians called "breaking bad news" to teach communication skills to all medical students at the Ludwig Maximilian University (LMU) in Munich [11]. In Heidelberg, the topic of "violence" is embedded in the curriculum across different specialties within the medical school [8]. In Ulm, there is a focus on "depression" and "pharmacotherapy" in cooperation with psychiatry [12].

In the German state of Baden-Wuerttemberg, *2 *of the 5 deans responsible for the medical curriculum are child psychiatrists and one is a paediatrician. A network of competence for medical education (*Kompetenznetz Lehre in der Medizin Baden-Wuerttemberg*) connects the 5 medical schools Freiburg, Heidelberg, Mannheim, Tuebingen, and Ulm. The focus is on academic didactics in medicine, with the faculty members at each medical school focusing on a separate area: "examination and standards in examination," "e-learning in medicine," "evaluation of teaching," "practical year," and "preparation for final examination." The practical year is a pre-degree internship year with 3 rotations. Contracts regulate a "collective organizational structure," "joint projects," "integrated quality assurance," "support by the Federal Ministry of Education," and "long-term financial funding of the medical schools [12-14].

On an international level, examples of collaboration are those between the medical schools of Heidelberg, and more recently also the LMU Munich, with Harvard Medical International (since 2008 Partners Harvard Medical International). New concepts of education have been developed and implemented through ongoing training of faculty and through the exchange of students [15].

## Discussion

The focus of the present survey was on teaching CAP to medical students in Austria, Germany, and the German-speaking part of Switzerland. A short questionnaire provided the opportunity to reach the respondents quickly and without their having to sacrifice too much time. But this type of survey also has drawbacks because some questions and answers may be imprecise. In the responses to the questionnaires, sometimes questions were left unanswered or the information provided was ambiguous. From the Web sites of the departments we could determine that in addition to medical students most departments of CAP also teach students from other faculties, such as psychology, pedagogy, and law. Some departments have excellent Web sites and it was easy to get appropriate additional information there (see Table 2 for examples of good Web sites).

**Table 2:** Web sites from some of the participating medical schools.^a^

Medical School	Web Site
Basel	http://www.upkbs.ch/apps/page.asp?Q=LehreForschung&menutab=5
Bern	http://www.gef.be.ch/site/index/upd/upd-forschung-lehre.htm
Hamburg	http://www.uke.de/kliniken/kinderpsychiatrie/
Heidelberg	http://www.klinikum.uni-heidelberg.de/Lehrveranstaltungen.99986.0.html
Muenster	http://kinderpsychiatrie.klinikum.uni-muenster.de/index.php?id=1951
Munich, LMU^b^	http://www.kjp.med.uni-muenchen.de/lehre.php
Ulm	http://www.uniklinik-ulm.de/struktur/kliniken/kinder-und-jugendpsychiatriepsychotherapie/home/lehre/lehrveranstaltungen.html
Wuerzburg	https://www-sbhome1.zv.uni-wuerzburg.de/qisserver/rds?state=wtree&search=1&trex=step&root120091=8125|8936|9013|8584&P.vx=kurz

^a^In German (not available in English); all accessed December 1, 2009.

^b^LMU: Ludwig Maximilian University Munich.

In the survey of European medical schools, the response rate was 48% (159 of 331) [3]. In the Australian survey it was 80% (12 of 15) [4], and in the United Kingdom survey it was 96% after 3 mailings (28 of 29) [16]. In our survey, the response rate of 100% can be regarded as an indicator of the high level of commitment of the departments of CAP. The results of the survey demonstrate that the program directors are making an effort to improve education by achieving more active involvement of the students. Diversifying teaching methods in CAP is a means of expanding educational opportunities and establishing the subject as a fixed component of the curriculum for medical students. Koch and Resch commented that "Since 2001, at the Heidelberg medical school (and some others as well) CAP has gained greater importance within the new curriculum and courses are now required" [8].

In the present study we found that medical education in CAP in the 3 countries surveyed is at different stages of development. In Austria, the process of CAP developing an identity of its own is at a relatively early stage, with interdisciplinary cooperation just beginning. In Switzerland, child psychiatry is integrated into the psychiatry curriculum and thus CAP is required in undergraduate medical education. In Germany, the situation is somewhere in between. Compared to the findings for German-speaking countries from 2000 [3], there is now a stronger commitment to teaching CAP. There is wide variation, with a core group of departments striving to have CAP become part of the required curriculum and to raise the overall standard of medical education.

The student/teacher ratio is an essential structural component in the organization of topics to be taught. A limitation of the present study is the lack of information on the number of CAP staff and how many students they teach.

In the review by Sawyer et al. on teaching activities [1], the most frequently reported amount of time allocated to teaching CAP was 12 to 24 hours in the course of medical school, with a wide range in all publications. The amount of time considered necessary by the participants in the European survey by Kálmán et al. [3], which included the German-speaking countries, was 35 hours, and an optimistic estimate was 150 hours.

The average of 34 hours per semester indicated by 21 of the 33 departments participating in the present study approximates the estimate for sufficient teaching time given by Kálmán et al. and can be understood as an indicator of a development towards increased participation of CAP in teaching medical students in recent years. The finding that at least 79% (n = 26) of the respondents in the present study are involved in teaching required courses for medical students is better than what we had expected.

We don't have information on teaching time from the students' perspective. It would be of interest to know how long and how often medical students are given the opportunity in required courses to acquire knowledge and skills in CAP.

### Quality Issues: Evaluation

In Cottrell's view [16], the inclusion of questions on CAP in final medical examinations is essential. In the European survey [3], 86% of the departments had an examination in CAP. Grades in CAP were given in 34% of these departments. The author concluded that "examination techniques provided a good measure of knowledge. But two thirds of the participants thought that they were ineffective for assessing skills and attitudes."

In the German-speaking countries, the assessment of student performance differs markedly among the different departments of CAP. The most common way of assessing the students' knowledge is a written exam, or rather some questions as part of the written exam in adult psychiatry. Some departments give oral examinations, use multiple-choice tests, do testing in the context of the OSCE, or even use standardized patients. But there are also departments that have no examination at all or the respondents provided no information on this topic. With an assessment ahead, students would have an incentive to participate actively. From the missing data we got the impression that those departments of CAP that are most involved in teaching medical students seem to be most willing to give precise details about the amount of time they teach and the kind of examination they give. A standardization of the examination across medical schools would be desirable to guarantee a predefined level of knowledge and skills in CAP and also to strengthen the position of CAP within medical schools.

Since evaluation is an important factor in the quality management of education in general, it would be advisable to get feedback on teachers from the students' perspective and also from that of the medical school. In the present survey, in addition to responding to the questions asked, 2 of the participants reported that their courses were evaluated by students. Lempp et al. [17] conducted a survey among 1031 students at 10 German universities attending their first lecture in CAP. The students expressed great interest in the field of CAP and assigned the specialty a high degree of relevance for their future work as physicians. The lecture topics rated as most relevant were presentation of patients (71%), knowledge about diseases (73%), skills (61%), and differentiation between normal and abnormal (59%). Of the 1031 students participating, 67% were women. In the present survey, only 33% of the staff were women. This suggests that gender issues relating to communication skills and career planning should be incorporated into the curriculum.

In Germany there is a ranking by medical school of students' examination results at the end of their medical studies. The allocation of resources for teaching by the ministries of science and education is affected by the quality of teaching, and hence there is a strong incentive to provide the students with effective ways of learning.

As measured by the number of publications, research on medical education seems to have a low priority. In a survey on research activities in Germany in the field of CAP between 2003 and 2008 [18], 8 articles about undergraduate education were found, which is not too different from the 18 identified by Sawyer et al. [1] for the period of 1970 to 2007. But among other things publications provide an opportunity to reflect on and better structure one's own teaching activities and hence to improve their quality. Furthermore, research is needed, for example, to develop new interdisciplinary modules and better ways of assessing communication skills.

### The Narrow Perspective: Electives

Regarding elective subjects, only 3 of the 7 departments responding to this question made precise statements. Some said that there were optional seminars without indicating whether these were part of the curriculum. There is no clarity about how many students actually receive any education in CAP, and if they do, how much.

In small groups the closer contact of the CAP staff with the students provides the opportunity to mediate skills more effectively than in large groups [19]. For the students, it is interesting and stimulating to have personal contact with patients. Skills in interviewing should be taught parallel to such contact. Optional courses are attractive for both the students interested in CAP and their teachers and make it easier to win young academics for the specialty of CAP even before the end of medical school.

Martin et al. [20] summarize current educational activities in CAP as "too little too late." Under the heading of "early and often" they present the new curriculum at Yale University School of Medicine, beginning with an early exposure of students to normal child development in connection with clinical problems and continuing far beyond the final examination to hiring young physicians as clinicians and as researchers. Our survey shows that in CAP becoming involved in teaching is no longer an isolated attempt, but has become a broader movement.

### A Broader Perspective: General Practice

An overarching goal of teaching medical students is to prepare them for general practice. To reach every student, CAP needs to cooperate with partners from other specialties. With the goal of joint teaching it is necessary to coordinate the different building blocks in the context of the whole curriculum, to avoid duplication, and to ensure that the teaching topics regarded as essential are covered. Active cooperation should take place between CAP and adult psychiatry, pediatrics, and psychosomatic medicine.

Longitudinal modules offer an opportunity for students to learn about subjects of general interest such as pharmacotherapy, the assessment of scientific studies, and the life cycle of normal development, and especially to have training in communication skills.

## Conclusions

The following steps can be taken to improve education in CAP for undergraduate medical students:

• Introduction of elective courses to help attract interested students to CAP as a career and to facilitate the development of skills through interactive teaching methods

• Involvement of CAP in required courses for all students

◦ through cooperation with psychiatry and psychosomatic medicine for psychopathology;

◦ through cooperation with pediatrics for development and family orientation

• Introduction of a longitudinal approach.

◦ The acquisition of communication skills lends itself to being incorporated into such an approach in cooperation with general medicine, internal medicine, surgery, public health, and other departments.

◦ Normal development in relationship to pathology in childhood can be taught in cooperation with medical psychology and pediatrics.

• Cooperation within medical schools and building networks among the different departments of child and adult psychiatry nationally and also internationally will contribute to standardizing goals and teaching methods and hence improving education for medical students.

In contrast to what Sawyer et al. [1] found, there is now broad agreement on global educational objectives in CAP on a national and international level. Moreover, the Swiss and German catalogues of learning objectives for undergraduate medical students are much more detailed than those proposed by Sawyer. For symptoms and diseases, learning objectives are structured by subject and by level of competence to be attained. Furthermore, the curriculum leaves room for local solutions. Finally, there is a trend towards more involvement of academic departments of CAP in teaching medical students, making CAP more attractive and strengthening its position as a medical specialty.

## Competing interests

The authors declare that they have no competing interests.

## Authors' contributions

RF conceived the survey. RF and FF collected data, described results and wrote the manuscript in equal parts. Both authors read and approved the final manuscript.

## Appendix 1

Questionnaire

sent to the 33 departments of CAP

(translation from the original German)

Dear............,

A recent article in the Journal of the American Academy of Child and Adolescent Psychiatry dealt with teaching child and adolescent psychiatry to undergraduate medical students (Sawyer MG, Giesen F, Walter G. 2008, 47:139-147). I would like to do a survey about teaching goals and methods for medical students in German-speaking countries and I would be most grateful for your cooperation. Please either answer the following questions yourself or ask someone in your department who is involved in teaching to reply.

What are teaching goals for (all) medical students? Please rank in order of importance.

Knowledge

• Normal development of children

• Knowledge about diagnosis and treatment of psychiatric disorders in children

• Other

Skills

• Assessment of children and adolescents

• Assessment of families

• Other

Which teaching methods do you use?

• Lectures

• Case presentations

• Other

Are you involved in teaching required courses for medical students?

• Yes

• No

If yes: How many hours per semester does your department spend on teaching such courses?

Is there cooperation with other specialities?

• Pediatrics

• Psychiatry

• Neurology

• Psychosomatic medicine

• Other

In what way? Please describe.

Are teaching goals assessed?

• Yes

• No

In what way? Please describe.

Thank you for your cooperation.

A stamped, self-addressed envelop is enclosed. I will send you a summary of the survey results.

Sincerely,
